# Development and validation of a nomogram for predicting cancer-specific survival in patients with Wilms' tumor

**DOI:** 10.7150/jca.32741

**Published:** 2019-08-28

**Authors:** Zhenyu Pan, Haisheng You, Qingting Bu, Xiaojie Feng, Fanfan Zhao, Yuanjie Li, Jun Lyu

**Affiliations:** 1 Clinical Research Center, The First Affiliated Hospital of Xi'an Jiaotong University, Xi'an, Shaanxi, China; 2 School of Public Health, Xi'an Jiaotong University Health Science Center, Xi'an, Shaanxi, China; 3 Department of Pharmacy, The Affiliated Children Hospital of Xi'an Jiaotong University, Xi'an, Shaanxi, China; 4 Department of Pharmacy, The First Affiliated Hospital of Xi'an Jiaotong University, Xi'an, Shaanxi, China; 5 Department of Genetics, Northwest Women's and Children's Hospital, Xi'an, Shaanxi, China; 6 Department of Human Anatomy, Histology and Embryology, School of Basic Medical Sciences, Xi'an Jiaotong University Health Science Center, Xi'an, Shaanxi, China

**Keywords:** Wilms' tumor, nomogram, predict, survival

## Abstract

**Purpose**: The objective of this study was to develop and validate a nomogram for predicting the cancer-specific survival (CSS) in patients with Wilms' tumor (WT).

**Methods**: Patients with WT diagnosed between 2002 and 2015 in the Surveillance, Epidemiology, and End Results (SEER) database were divided randomly into training and validation cohorts in this study. Multivariate Cox regression analysis was used to screen variables. A Cox proportional-hazards regression model and a nomogram were constructed based on variables that significantly affected the CSS in the training cohort. The nomogram for distinguishing and predicting the CSS was evaluated using the concordance index (C-index), the area under the time-dependent receiver operating characteristic curve (AUC), and calibration plots.

**Results**: In total, 1631 patients from the SEER database were enrolled, with 1141 categorized into the training cohort and 490 into the validation cohort. All significant variables associated with CSS—age, the number of examined lymph nodes, SEER stage, and tumor size—were included in the nomogram. The C-index values of the nomogram in the training and validation cohorts were 0.746 and 0.703, respectively. The 3-, 5-, and 10-year AUCs were 0.755, 0.749, and 0.724, respectively, in the training cohort, and 0.718, 0.707, and 0.718 in the validation cohort. The calibration plots indicated the nomogram could accurately predict the 3-, 5-, and 10-year CSS.

**Conclusions**: We have developed and validated the first nomogram for predicting the survival of WT patients. The nomogram is a reliable tool for distinguishing and predicting the CSS in patients with WT. Information provided by the nomogram may help to improve the clinical practices related to WT.

## Introduction

Wilms' tumor (WT) is the most-common type of pediatric renal tumor, constituting about 95% of all pediatric renal cancers 1 and 5% of pediatric cancers 2. Although more than 90% of WT patients receiving the current multimodal therapy exhibit long-term survival 3, the prognosis of patients is still a major research focus because future better treatment prescriptions need to be based on knowledge of the prognosis risk of patients. Some prognostic factors—including age, tumor size 4-6, Surveillance, Epidemiology, and End Results (SEER) stage 7, and the number of examined lymph nodes (LNs) 8—have been found to significantly affect survival. However, faced with these unconsolidated factors, none of studies incorporated them to accurately predict the prognosis of patients with WT. It is therefore necessary to integrate multiple prognostic factors into an easy-to-use predictive system to better stratify the prognosis of patients with WT.

A nomogram is a predictive tool that appears as a simple graph based on a statistical predictive model 9. It can be used to calculate the probability of a clinical event by considering the prognostic weight of each factor. Nomograms have been widely used in recent years for predicting the survival in various cancers 9. However, to the best of our knowledge, no nomograms for patients with WT have been reported.

This study aimed to incorporate some important factors obtained from analyzing data from the SEER database in the development and validation of a nomogram for predicting the cancer-specific survival (CSS) of patients with WT.

## Materials and Methods

### Patients and variables

Patients with WT from the SEER database were analyzed from 1973 to 2015 using the SEER*Stat software (version 8.3.5) 10. Patients inclusion criteria: patients were diagnosed with WT (histological diagnostic code 8960 in the third edition of the International Classification of Diseases for Oncology). Patients exclusion criteria: (1) patient was diagnosed before 2002; (2) patients with overlapping data; (3) there are missing data in the following variables: age (year), sex, race, the number of examined LNs, SEER stage, tumor laterality, metastasis, radiation, chemotherapy, tumor size (millimeter, mm), follow-up time, and cause-specific death. According to the SEER database, in SEER stage, a localized tumor is defined as one “limited to the organ in which it began, without evidence of spread,” a regional tumor has “spread beyond the primary site to nearby lymph nodes or organs and tissues”, and a distant tumor has “spread from the primary site to distant organs or distant lymph nodes”. This study was performed in accordance with Declaration of Helsinki and was approved by the institutional review board of the First Affiliated Hospital of Xi'an Jiaotong University.

### Statistical analysis

The included patients were randomized into a training cohort and a validation cohort at a ratio of 7:3. Multivariate Cox regression analysis was performed to identify variables that significantly affect CSS. The model for the nomogram was constructed using the significant variables.

The nomogram was validated by measuring discrimination and calibration in both the training and validation cohorts. Discrimination was evaluated using the concordance index (C-index) 11 with a bootstrap approach involving 500 resamples and the area under the time-dependent receiver operating characteristic curve (AUC) 12,13. A C-index or AUC of 0.5 indicates a discrimination ability that is no better than chance, and one of 1.0 indicates a perfect discrimination ability 14. The calibration curves were applied using a bootstrap approach with 500 resamples to compare the predicted CSS with the CSS observed in the study. The calibration curve is along the 45-degree line of the calibration plot in a perfect calibration model, which indicates that the predicted CSS probabilities are identical to the actual ones.

All statistical analyses were conducted using SPSS (version 24.0, SPSS, Chicago, IL, USA) and R software (version 3.4.3; http://www.r-project.org/). A P value of ≤0.05 was considered statistically significant.

## Results

### Patient characteristics

The study included 1631 eligible patients with WT diagnosed between 2002 and 2015, and categorized 1141 into the training cohort and 490 into the validation cohort. In the total cohort, the median age was 3 years, the median number of examined LNs was 3, the median tumor size was 110 mm, and most of the patients were female (54%), white (76.8%), and had a tumor that was localized (44.8%), unilateral (93.5%), and without metastasis (79.2%). Almost half (45.9%) of the patients received radiation, and while 91.4% received chemotherapy. The characteristics in the training and validation cohorts were similar to those in the total cohort (Table 1).

### Nomogram construction

**Figure 1:**
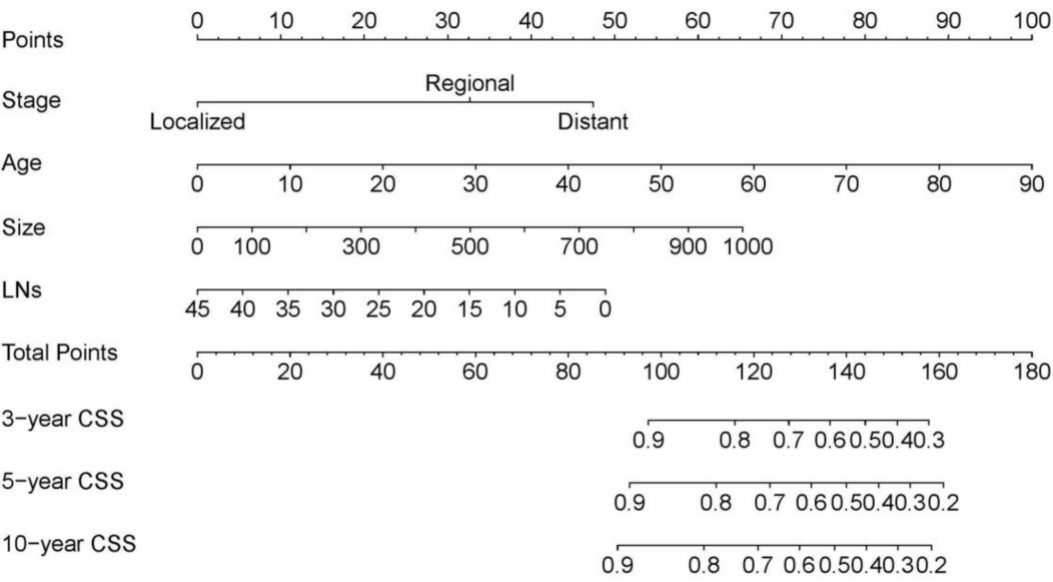
The nomogram predicting CSS in patients with WT. Each factor was given a point on the basis of the nomograms. The total points were obtained by adding the given points of all factors. The estimated 3-, 5-, and 10-year probabilities of CSS of the individual patient can be easily obtained from the nomogram based on the total points.

Data on variables including age, sex, race, the number of examined LNs, SEER stage, tumor laterality, metastasis, radiation, chemotherapy, and tumor size were collected in the training cohort. In the multivariate analysis, age, the number of examined LNs, SEER stage, and tumor size were significantly associated with CSS (Table 2), and so the model was constructed based on these four variables (Table 3). This model for the training cohort was then used to construct a nomogram for predicting the 3-, 5-, and 10-year CSSs (Figure 1). Each variable is given a point on the nomogram, and the total point can be obtained by adding the scores. The total point corresponded to CSS probabilities which could be indicated by the nomograms.

### Nomogram validation

**Figure 2:**
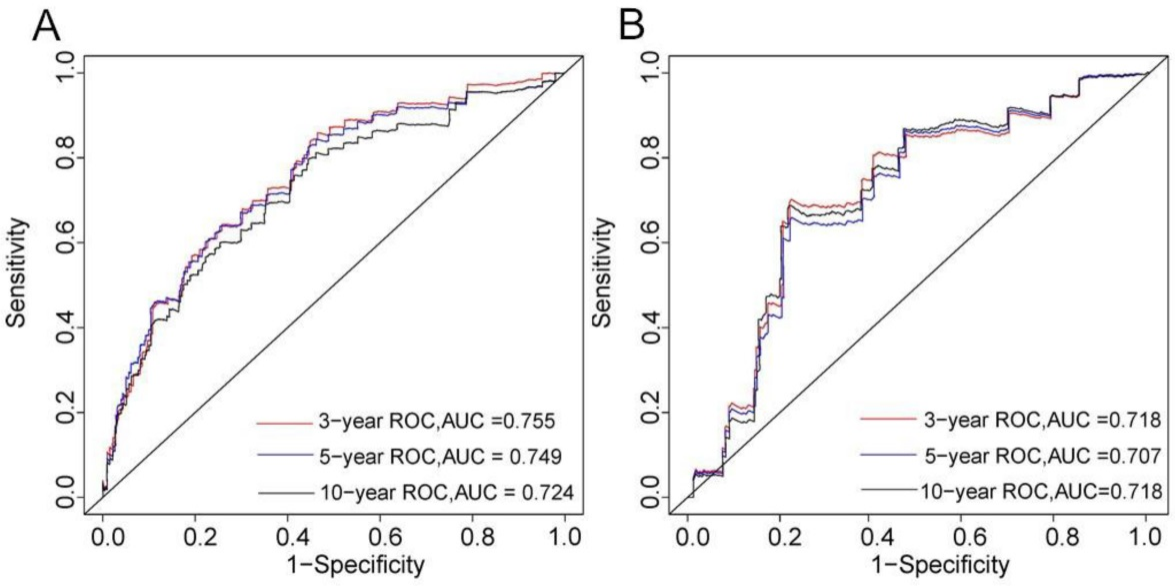
3-, 5-, and 10-years ROC curves in training (A) and validation cohorts (B) for validating nomogram model.

The C-index of the nomogram was 0.746 in the training cohort and 0.703 in the validation cohort. The 3-, 5-, and 10-year AUCs were 0.755, 0.749, and 0.724, respectively, in the training cohort (Figure 2A), and 0.718, 0.707, and 0.718 in the validation cohort (Figure 2B). These results indicated that the discrimination performance of the model was good in both the training and validation cohorts.

**Figure 3:**
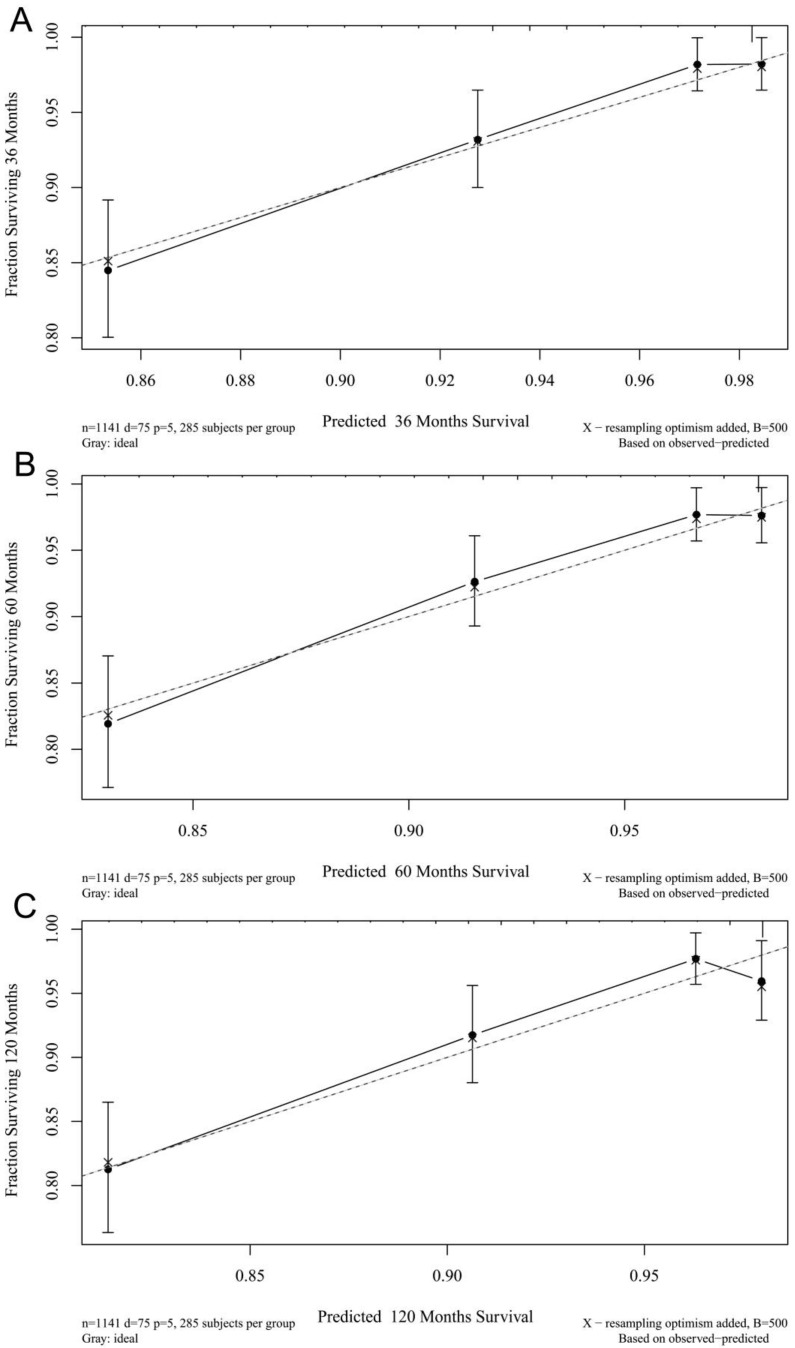
3- (A), 5- (B), and 10-years (C) calibration curves for probability of CSS nomogram construction in training cohort (Bootstrap = 500 repetitions).

**Figure 4:**
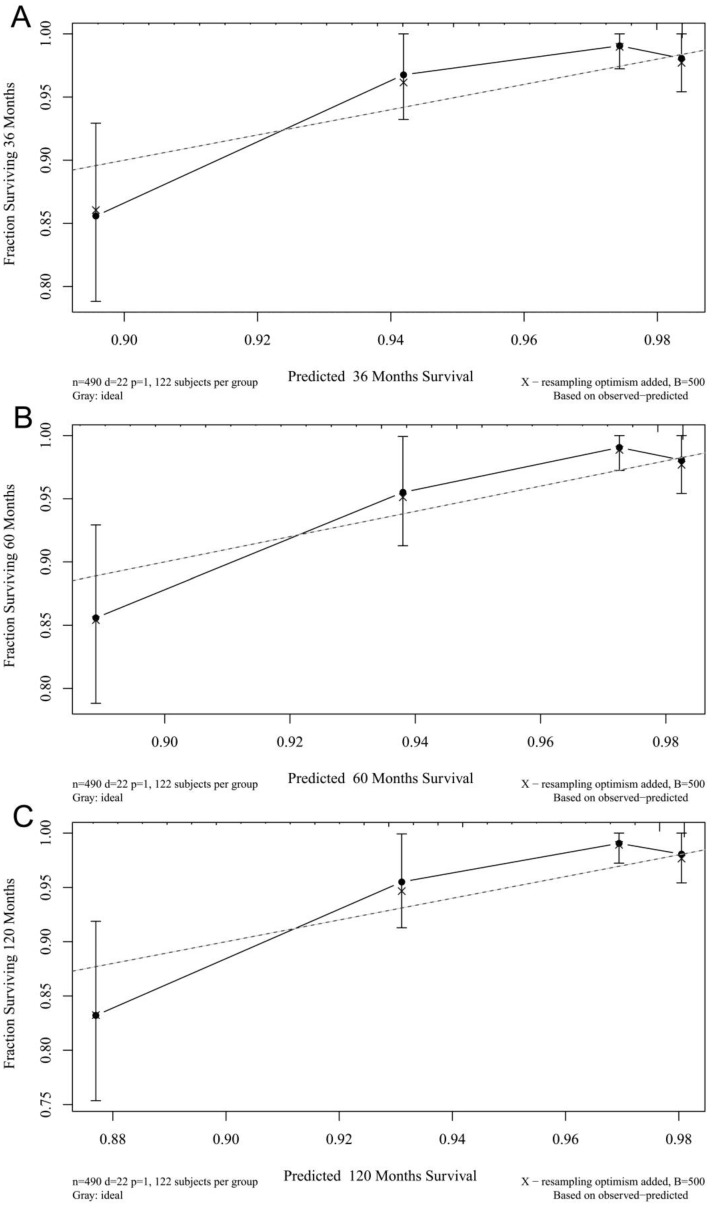
3- (A), 5- (B), and 10-years (C) calibration curves for probability of CSS nomogram construction in validation cohort (Bootstrap = 500 repetitions).

The calibration plots for the 3-, 5-, and 10-year CSSs indicated that there was good agreement between the actual observations and predictions made using the nomogram in both the training cohort (Figure 3) and the validation cohort (Figure 4).

## Discussion

WT is the most common primary renal malignancy occurring in childhood. Typically 4-7% of malignant tumors occur in children younger than 15 years, and 90% of renal tumors occurring in children are WT 15. About 500 children are diagnosed with WT annually in the United States, most of whom are younger than 5 years. The treatment of WT is one of the great success stories in modern medicine, with the overall survival rate of patients exceeding 90%. Although this is a remarkable achievement, the about 25% of all patients with WT were at higher risk and their overall survival rates remain below 90%. Moreover, the patients at lower risk may experience overtreatment, including excessive radiotherapy and chemotherapy. Therefore, a tool for distinguishing patient risk that is better than simple tumor staging is needed, because it would enable clinicians to apply more-precise and appropriate therapy. Factors including patient age, tumor size 4-6, loss of heterozygosity (LOH) at chromosomes 1p and 16q 16-18, LN density 19, and the number of examined LNs 8 were recently found to significantly impact the survival of patients with WT. It is challenging to construct a model by combining the useful factors for improving predictions of the prognosis of patients with WT. In this study we developed and validated a nomogram for predicting the CSS of patients with WT by incorporating some important factors identified in a Cox proportional-hazards model.

The source of patients for constructing the nomogram was the SEER database of the United States National Cancer Institute. The annual incidence of WT in children under 15 years of age **is** between 0.051 and 0.095 per 10,000, so there will be too few cases to construct or validate a model if we rely only on the patients at up to several hospitals. In contrast, the SEER database includes 18 registries covering approximately 28% of the population in the United States 20, and is the largest registry of information on cancer in the United States. This means that utilizing the database should provide a sufficient number of WT cases.

We excluded patients diagnosed prior to 2002 since the latest clinical trial conducted by the National Wilms Tumor Study Group (called NWTSG-5) was reported on in 2001 6. The treatments or other circumstances before 2002 may differ from those at present. A nomogram constructed that includes patients before 2002 is therefore not suitable for predicting present patients. This study analyzed a total of 1631 patients.

We chose the following 8 factors to construct the nomogram: age, sex, race, the number of examined LNs, SEER stage, tumor laterality, metastasis, radiation, chemotherapy, and tumor size. A good nomogram should include the minimum number of factors required to ensure good discrimination and calibration, and so this study applied multivariate Cox regression analysis to choose the most-important factors impacting the CSS of patients with WT. The final factors incorporated in the nomogram were age, the number of examined LNs, SEER stage, and tumor size; these four factors are easy to measure during diagnosis and treatment. The constructed nomogram was validated and evaluated based on discrimination (C-index and AUC) and calibration. It is widely known that a model has a relative good discrimination if its C-index and AUC exceed 0.7 21-23, and so the present results indicated that the nomogram we have constructed exhibits good discrimination and calibration. Clinicians can therefore use the nomogram to predict the CSS in WT patients based on the model employed.

It should be noted that there were several limitations in this study. First, some potentially important fact ors such as LOH at chromosomes 1p and 16q, the response to chemotherapy, and histology findings were not analyzed when constructing the nomogram because these factors are not available in the SEER database. Incorporating such important factors might further improve the discrimination and calibration of the model. Second, only “yes” or “no/unknown” information about radiotherapy and chemotherapy information can be obtained, rather than more-specific information such as the dose, type, and course of treatment. Third, only an internal validation of the nomogram was performed, and so the nomogram still needs to be externally validated using other populations with WT.

In summary, we have developed and validated a nomogram for predicting the CSS in patients with WT. To the best of our knowledge, this is the first nomogram for predicting the survival of WT patients. We have demonstrated that it shows good discrimination and calibration. This nomogram may be instructive to establish a better prediction model by considering some potentially important factors we cannot obtain in the SEER database and external validation.

## Figures and Tables

**Table 1:** Patient characteristics in the study.

	Total cohort	Training cohort	Validation cohort
Patients, n	1631	1141	490
Age (year), median (25th-75th percentile)	3(1-5)	3(1-5)	3(1-5)
Sex, n(%)			
Male	750(46.0)	532(46.6)	218(44.5)
Female	881(54.0)	609(53.4)	272(55.5)
Race, n(%)			
White	1252(76.8)	872(76.4)	380(77.6)
Black	281(17.2)	201(17.6)	80(16.3)
Other	98(6.0)	68(6.0)	30(6.1)
The number of examined LNs, median (25th-75th percentile)	3(1-7)	3(1-7)	3(1-7)
SEER stage, n(%)			
Localized	730(44.8)	504(44.2)	226(46.1)
Regional	516(31.6)	361(31.6)	155(31.6)
Distant	385(23.6)	276(24.2)	109(22.2)
Tumor laterality, n(%)			
Unilateral	1525(93.5)	1063(93.2)	462(94.3)
Bilateral	106(6.5)	78(6.8)	28(5.7)
Metastasis, n(%)			
Yes	340(20.8)	245(21.5)	95(19.4)
No	1291(79.2)	896(78.5)	395(80.6)
Radiation, n(%)			
Yes	748(45.9)	532(46.6)	216(44.1)
No	883(54.1)	609(53.4)	274(55.9)
Chemotherapy, n(%)			
Yes	1490(91.4)	1047(91.8)	443(90.4)
No	141(8.6)	94(8.2)	47(9.6)
Tumor size (mm), median (25th-75th percentile)	110(80-135)	105(76-134)	110(85-140)

**Table 2:** Multivariate Cox regression analysis based on all variables (training cohort).

	Hazard ratio (95%CI)	P value
Age	1.0349 (1.0091-1.061)	0.007780
Sex		
Male	Reference	
Female	1.0981 (0.6886-1.751)	0.694270
Race		
White	Reference	
Black	0.6533 (0.3328-1.283)	0.216111
Other	0.6365 (0.1546-2.620)	0.531408
The number of examined LNs	0.9523 (0.9087-0.998)	0.040785
SEER stage		
Localized	Reference	
Regional	4.1152 (1.8659-9.076)	0.000456
Distant	2.3683 (0.4872-11.512)	0.285211
Tumor laterality		
Unilateral	Reference	
Bilateral	1.6834 (0.7425-3.817)	0.212384
Metastasis		
No	Reference	
Yes	3.5143 (0.8294-14.890)	0.087995
Radiation		
Yes	Reference	
No	0.8901 (0.5162-1.535)	0.675360
Chemotherapy		
Yes	Reference	
No	2.4324 (0.9190-6.438)	0.073485
Tumor size	1.0033 (1.0000-1.006)	0.047171

**Table 3:** The Cox proportional hazard regression model for nomogram based on age, the number of examined LNs, SEER stage, and tumor size.

	Coefficient	Hazard ratio (95%CI)
Age	0.044653	1.0457 (1.0228-1.069)
The number of examined LNs	-0.043743	0.9572 (0.9145-1.002)
SEER stage		
Localized	Reference	Reference
Regional	1.315432	3.7264 (1.8289-7.592)
Distant	1.909331	6.7486 (3.4064-13.370)
Tumor size	0.002633	1.0026 (0.9993-1.006)

## References

[1] Ali AN, Diaz R, Shu HK (2012). A Surveillance, Epidemiology and End Results (SEER) Program Comparison of Adult and Pediatric Wilms' Tumor. Cancer.

[2] Friedman AD (2013). Wilms tumor. Pediatr Rev.

[3] Cotton CA, Peterson S, Norkool PA (2009). Early and late mortality after diagnosis of wilms tumor. J Clin Oncol.

[4] Metzger ML, Dome JS (2005). Current therapy for Wilms' tumor. Oncologist.

[5] Sredni ST, Gadd S, Huang CC (2009). Subsets of very low risk Wilms tumor show distinctive gene expression, histologic, and clinical features. Clin Cancer Res.

[6] Green DM, Breslow NE, Beckwith JB (2001). Treatment with nephrectomy only for small, stage I/favorable histology Wilms' tumor: a report from the National Wilms' Tumor Study Group. J Clin Oncol.

[7] Gratias EJ, Dome JS (2008). Current and Emerging Chemotherapy Treatment Strategies for Wilms Tumor in North America. Paediatr Drugs.

[8] Zhuge Y, Cheung MC, Yang R (2011). Improved survival with lymph node sampling in Wilms tumor. J Surg Res.

[9] Iasonos A, Schrag D, Raj GV (2008). How To Build and Interpret a Nomogram for Cancer Prognosis. J Clin Oncol.

[10] SEER Program. SEER*Stat Database: Incidence - SEER 18 Regs Custom Data (with additional treatment fields), Nov 2017 Sub (1973-2015 varying) - Linked To County Attributes - Total U.S, 1969-2016 Counties, National Cancer Institute, DCCPS, Surveillance Research Program, released April 2018, based on the November 2017 submission. Accessed 28 June 2018.

[11] Wolbers M, Koller MT, Witteman JC (2009). Prognostic models with competing risks: methods and application to coronary risk prediction. Epidemiology.

[12] Hanley JA, McNeil BJ (1982). The meaning and use of the area under a receiver operating characteristic (ROC) curve. Radiology.

[13] Deng H, Qi X, Zhang Y (2017). Diagnostic accuracy of contrast-enhanced computed tomography for esophageal varices in liver cirrhosis: a retrospective observational study. J Evid Based Med.

[14] Balachandran VP, Gonen M, Smith JJ (2015). Nomograms in oncology: more than meets the eye. Lancet Oncol.

[15] Davidoff AM (2009). Wilms' tumor. Curr Opin Pediatr.

[16] Dome JS, Fernandez CV, Mullen EA (2013). Children's Oncology Group's 2013 blueprint for research: Renal tumors. Pediatr Blood Cancer.

[17] DomeJS PerlmanEJ, GrafN (2014). Riskstratification for Wilms tumor: Current approach and future directions.

[18] Bu Q, He H, Fan D (2018). Association between loss of heterozygosity of chromosome 16q and survival in Wilms' tumor: A meta-analysis. Pathol Res Pract.

[19] You H, Yang J, Liu Q (2018). The impact of the lymph node density on overall survival in patients with Wilms' tumor: A SEER analysis. Cancer Manag Res.

[20] Wang S, Gao WC, Chen SS (2017). Primary site surgery for metastatic adrenocortical carcinoma improves survival outcomes: an analysis of a population-based database. Onco Targets Ther.

[21] Ruixue Ma, Yuegui Jiang, Xinghua Ma (2016). Prognostic analysis of oropharyngeal cancer by Nomogram. Modern Oncology.

[22] Jia Y, Li H, Wang J (2019). Spectrum structures and biological functions of 8-mers in the human genome. Genomics.

[23] Kobayashi S, Hiwasa T, Arasawa T (2018). Identification of specific and common diagnostic antibody markers for gastrointestinal cancers by SEREX screening using testis cDNA phage library. Oncotarget.

